# Effects of post-acute COVID-19 syndrome on cerebral white matter and emotional health among non-hospitalized individuals

**DOI:** 10.3389/fneur.2024.1432450

**Published:** 2024-08-06

**Authors:** Nathan W. Churchill, Eugenie Roudaia, J. Jean Chen, Allison Sekuler, Fuqiang Gao, Mario Masellis, Benjamin Lam, Ivy Cheng, Chris Heyn, Sandra E. Black, Bradley J. MacIntosh, Simon J. Graham, Tom A. Schweizer

**Affiliations:** ^1^Brain Health and Wellness Research Program, St. Michael’s Hospital, Unity Health Toronto, Toronto, ON, Canada; ^2^Keenan Research Centre for Biomedical Science of St. Michael’s Hospital, Unity Health Toronto, Toronto, ON, Canada; ^3^Physics Department, Toronto Metropolitan University, Toronto, ON, Canada; ^4^Rotman Research Institute, Baycrest Academy for Research and Education, Toronto, ON, Canada; ^5^Department of Medical Biophysics, University of Toronto, Toronto, ON, Canada; ^6^Institute of Biomedical Engineering, University of Toronto, Toronto, ON, Canada; ^7^Department of Psychology, University of Toronto, Toronto, ON, Canada; ^8^Department of Psychology, Neuroscience and Behaviour, McMaster University, Hamilton, ON, Canada; ^9^Hurvitz Brain Sciences Program, Sunnybrook Research Institute, Toronto, ON, Canada; ^10^Division of Neurology, Department of Medicine, Sunnybrook Health Sciences Centre, University of Toronto, Toronto, ON, Canada; ^11^Evaluative Clinical Sciences, Sunnybrook Research Institute, Toronto, ON, Canada; ^12^Integrated Community Program, Sunnybrook Research Institute, Toronto, ON, Canada; ^13^Department of Medicine, University of Toronto, Toronto, ON, Canada; ^14^Department of Medical Imaging, University of Toronto, Toronto, ON, Canada; ^15^Physical Sciences Platform, Sunnybrook Research Institute, Toronto, ON, Canada; ^16^Computational Radiology and Artificial Intelligence Unit, Division of Radiology and Nuclear Medicine, Oslo University Hospital, Oslo, Norway; ^17^Faculty of Medicine (Neurosurgery), University of Toronto, Toronto, ON, Canada

**Keywords:** white matter, COVID-19, emotions, DTI, DKI, NODDI

## Abstract

**Introduction:**

Post-acute COVID syndrome (PACS) is a growing concern, given its impact on mental health and quality of life. However, its effects on cerebral white matter remain poorly understood, particularly in non-hospitalized cohorts. The goals of this cross-sectional, observational study were to examine (1) whether PACS was associated with distinct alterations in white matter microstructure, compared to symptom-matched non-COVID viral infection; and (2) whether microstructural alterations correlated with indices of post-COVID emotional health.

**Methods:**

Data were collected for 54 symptomatic individuals who tested positive for COVID-19 (mean age 41 ± 12 yrs., 36 female) and 14 controls who tested negative for COVID-19 (mean age 41 ± 14 yrs., 8 female), with both groups assessed an average of 4–5 months after COVID testing. Diffusion magnetic resonance imaging data were collected, and emotional health was assessed via the NIH emotion toolbox, with summary scores indexing social satisfaction, well-being and negative affect.

**Results:**

Despite similar symptoms, the COVID-19 group had reduced mean and axial diffusivity, along with increased mean kurtosis and neurite dispersion, in deep white matter. After adjusting for social satisfaction, higher levels of negative affect in the COVID-19 group were also correlated with increased mean kurtosis and reduced free water in white matter.

**Discussion:**

These results provide preliminary evidence that indices of white matter microstructure distinguish PACS from symptomatic non-COVID infection. Moreover, white matter effects seen in PACS correlate with the severity of emotional sequelae, providing novel insights into this highly prevalent disorder.

## 1 Introduction

Severe Acute Respiratory Syndrome Coronavirus-2 (SARS-CoV-2) and the associated Coronavirus Disease 2019 (COVID-19) represent an ongoing health crisis (1), with long-lasting health effects among survivors. Of growing concern is post-acute COVID syndrome (PACS), in which symptom impairments last well beyond the acute phase of infection; typical definitions involve COVID-related symptoms that persist over 12 weeks post-infection (2, 3). This condition is highly prevalent, with persistent symptoms reported in over 30% of COVID-19 survivors (4). Lasting issues related to mood and psychological distress are also common features of PACS (5), raising concerns about emotional health and long-term quality of life. The biological processes that underlie PACS are understudied, however, particularly for non-hospitalized cohorts. Evidence continues to mount that the brain is vulnerable to COVID-19 (6), but it is uncertain to what extent persistent symptoms represent injury sustained during acute infection, persistent inflammatory effects, and/or psychological sequelae.

A better understanding of PACS pathophysiology is needed to characterize this disorder. In this respect, cerebral white matter is of interest, as it is the anatomical substrate supporting inter-regional communication and it is vulnerable to diffuse disease processes. The heterogeneity and multi-domain nature of post-COVID neurological symptoms (7) further supports the presence of diffuse white matter pathophysiology as a contributing factor (8, 9). Such post-COVID changes in white matter microstructure can be measured using diffusion-weighted magnetic resonance imaging (dMRI), which obtains brain images sensitive to the local diffusion properties of water. DMRI techniques have been shown to be sensitive to a variety of disease processes (10), including inflammation, axonal degeneration, demyelination and edema.

Standard dMRI techniques such as diffusion tensor imaging (DTI) quantify water diffusion properties of fractional anisotropy (FA), reflecting the directionality of water movement, and mean diffusivity (MD), reflecting the rate of movement; the latter may be split into axial diffusivity (AD) and radial diffusivity (RD) components, reflecting movements parallel and perpendicular to the primary axis of diffusion (11). This approach models tissue water as a single compartment with anisotropic Gaussian diffusion. More advanced models are also used, in which images with different diffusion weightings are acquired. This includes diffusion kurtosis imaging (DKI) (12), which estimates mean kurtosis (MK), reflecting departure from Gaussianity. Other techniques model multiple tissue water compartments, such as neurite orientation density and dispersion imaging (NODDI) (13). This technique estimates the fractional intracellular water volume (V_IC_) and isotropic free water volume (V_ISO_), along with an orientation dispersion index (ODI) measuring the geometric organization of neurites. Research is increasingly using dMRI to study COVID-19, but much remains unknown. Studies have used dMRI to assess individuals who have recovered from COVID-19 in hospitalized and non-hospitalized cohorts (14–18), along with cohorts with persistent post-COVID symptoms (19), usually compared to healthy uninfected controls. Less is known about dMRI effects among non-hospitalized PACS cohorts, and it is presently unclear to what extent such effects differ from non-COVID viral infection. This is an important issue to investigate, in order to establish neural involvement that is specific to COVID-19, as opposed to a reflection of general systemic inflammation (20, 21). Moreover, the relationship of dMRI with clinical measures of emotional health has been understudied in PACS, making it unclear to what extent white matter pathophysiology underlies these highly prevalent issues (5).

The present study investigated these knowledge gaps using dMRI data collected from self-isolating individuals who tested positive for SARS-CoV-2 and subsequently experienced persistent symptoms. They were compared to a control group that had cold or flulike symptoms and tested negative for SARS-CoV-2, with both groups imaged an average of 4–5 months after testing for COVID-19 infection. For the COVID-19 group, associations were further tested between dMRI and measures of emotional health, collected via the National Institutes of Health (NIH) Toolbox (22), a computerized platform that assesses behavioral and neurologic function. It was hypothesized that, despite both groups reporting post-infection symptoms, the COVID-19 group would show increased FA, MK and ODI, and reduced MD, AD, RD and V_IC_, as a consequence of COVID-related neural injury (15, 16, 23), and that the effects would be greater for those in the COVID-19 group with poorer indices of emotional health.

## 2 Materials and methods

### 2.1 Study participants

Study participants were recruited through multiple sources, including the emergency department at a single Canadian hospital, physician referral and community advertisements, following the protocol outlined in (24). Candidate participants were identified via electronic records and contacted by phone or email to verify eligibility and to obtain consent to participate. They were eligible if between 20 and 75 years of age and living independently, and they had documented evidence of a positive or negative COVID-19 diagnosis, obtained via nasopharyngeal or oropharyngeal swab with real-time reverse transcription polymerase chain reaction (PCR) testing, conducted at a provincially-approved facility (25). They were excluded from the study if they previously had a diagnosis of dementia, neurologic disorder, severe psychiatric illness, traumatic brain injury or ongoing unstable cardiovascular disease, or contraindication to MRI (e.g., claustrophobia or ferromagnetic implant). Both COVID-positive participants and COVID-negative controls were assessed a minimum of 14 days post-infection to ensure that they were non-infectious, and they were required to be symptomatic at the time of assessment (see section 2.3 below for further details of symptom assessment). The COVID-positive group was further required to have been non-hospitalized and to have self-isolated following a positive diagnosis. Recruitment and data collection were carried out in accordance with the Canadian Tri-Council Policy Statement 2 and institutional research ethics board, with participants giving free and written informed consent.

Participant recruitment and data collection was carried out between May 2020 and December 2021, which was predominantly during the initial “wild-type” infection wave (26). In addition, 62/68 participants were scanned prior to public vaccine availability in Ontario, which was initiated in April 2021 (27). Within the study cohort, 4 participants (all with COVID-19) were vaccinated prior to the study-relevant viral infection, and only 1 study participant (with COVID-19) had a previous lab-confirmed COVID-19 infection, identified 580 days prior to the study-relevant infection. Initial testing did not find these participants to be significant outliers in terms of demographics, clinical data or dMRI data, and thus were retained for further analysis.

### 2.2 Magnetic resonance imaging data

Participants were imaged at a single site using a 3 Tesla MRI system (Magnetom Prisma, Siemens Healthineers). Diffusion imaging involved three acquisitions of 2D echo-planar imaging data (2.5 mm isotropic voxels, obtained via 96 × 96 matrix with 60 oblique-axial slices, 62/4300 ms TE/TR, 30 diffusion encoding directions and 4 b0 scans, A> > P phase encoding); imaging was obtained for *b*-values of 700, 1,400 and 2,100 s/mm^2^. An additional pair of unweighted b0 images with reversed phase encoding (P> > A) were also collected prior to diffusion-weighted imaging to correct for spatial distortions. The data were processed using a hybrid pipeline that included FSL (FMRIB software library),^1^ DTI-TK^2^ and in-house software (see Appendix 1 for details). DTI parameters were obtained for each participant using the FSL *dtifit* program, including fractional anisotropy (FA), mean diffusivity (MD), axial diffusivity (AD) and radial diffusivity (RD), for each of the diffusion weightings. The set of diffusion weightings was also used to calculate mean kurtosis (MK) with *dtifit* and NODDI parameters were estimated using the Matlab toolbox.^3^ The latter parameter set included volume fractions of intra-cellular neurite water (V_IC_) and isotropic free water (V_ISO_), along with the orientation dispersion index (ODI) assessing the spatial organization of neurites. The diffusion parameter maps were warped into Montreal Neurological Institute (MNI) standard space and resampled to 3 mm isotropic voxel resolution, using DTI-TK’s tensor-based diffeomorphic alignment tools to register to the IIT Human Brain Atlas’ mean tensor template (28).

Subsequent voxel-wise analyses were restricted to a mask of regions with mean FA > 0.30, consisting of probable white matter. Before analysis, the resampled parameter maps were also spatially smoothed using a 3D Gaussian kernel with 6 mm full width at half maximum (FWHM), to reduce voxel noise and make analyses robust to minor spatial variability between participants. Outlier testing found no participants with abnormal head motion or abnormal diffusion signal changes, and only one participant (COVID-19) whose MK and V_ISO_ maps had abnormal values (suggesting poor fit), which were excluded from analysis. Post-hoc testing of head motion also found no significant confounding effects for the main study analyses (see Appendix 1 for details). In subsequent analyses, for DTI parameter maps, results are presented for *b* = 1,400 s/mm^2^ only, as minimal differences were seen in other acquisitions; whereas MK and NODDI parameter estimates were computed from all three *b*-value shells.

### 2.3 Analysis of clinical and demographic data

Participant demographics were tabulated including age, sex, years of education, days from symptom onset to imaging, and days from PCR test to imaging. Participants also completed a questionnaire evaluating symptom status for 9 items: fever, cough, sore throat, shortness of breath, fatigue, gastrointestinal issues, problems with smell/taste, headache and “other.” They reported whether each symptom (1) was absent, (2) had occurred but resolved, or (3) was currently ongoing. Symptoms were identified if onset was concurrent with, or subsequent to, the study-relevant viral infection and PCR test. Participants also completed the emotional health component of the NIH Toolbox (29). This computerized program provides a battery of questionnaires assessing emotions, attitudes and inter-personal relationships. Test scores are aggregated into domain-specific summary measures, including social satisfaction (SocSat), well-being (WelBei) and negative affect (NegAff), before conversion to demographic-adjusted normalized T-scores (mean = 50, SD = 10). NIH Toolbox data were not collected for four participants (1 control, 3 COVID-19), hence they were excluded from emotional health analyses.

After assessing the normality of demographic and clinical variables by comparing skewness and kurtosis values against a simulated null distribution (5,000 resamples), means and standard deviations were reported for measures that did not deviate from normality and medians with upper and lower quartiles were reported for those that did. For analyses of both clinical and neuroimaging data, bootstrapping was used to estimate the confidence intervals and *p*-values for effects of interest. This approach randomly resamples on participants with replacement (2000 iterations) and recomputes the test statistic of interest for each sample, thereby providing an empirical sampling distribution (30). This non-parametric technique provides a highly flexible approach to statistical inference, which does not depend on modeling assumptions common to many parametric models, such as the normality of residuals and homogeneity of variance; the latter is particularly important in cases of unbalanced group sizes (31). The frequency of ongoing and resolved individual symptoms was also reported, with bootstrapped 95% confidence intervals (95%CIs), along with average number of symptoms reported, for “ongoing” symptoms and for “combined” symptoms (ongoing plus resolved). A series of 2-sample bootstrap analyses (2000 iterations) tested for group differences in demographics (two-tailed), symptom reporting frequencies, symptom counts, and emotional summary scores. For the emotional scores, COVID-19 and control groups were further compared to the normative reference, via 1-sample bootstrap analyses of the difference from the normative mean (*T* = 50). Summary statistics were reported for all analyses, including group differences, their standard errors (SEs), bootstrap ratio values (BSR; this is a z-scored statistic of effect, calculated via the ratio of bootstrap mean/SE), along with two-tailed percentile *p*-values. Differences of means were reported for variables that did not deviate from normality, and differences of medians were reported for those that did, with significant effects identified at *p* < 0.05, unadjusted.

### 2.4 Effects of COVID-19 on dMRI data

For each dMRI parameter, voxel-wise analyses were performed assessing the mean difference between COVID-19 and control groups. Group differences were estimated using a general linear model (GLM), with covariates adjusting for age and sex, as these parameters may influence dMRI values (32–34). Significant voxels were identified based on the bootstrapping of group effects (2000 iterations), adjusted for multiple comparisons: voxels were retained where the 99.5%CI did not include zero (corresponding to a threshold of *p* < 0.005, two-tailed), followed by cluster-size thresholding at *p* < 0.05 using Analysis of Functional NeuroImages (AFNI) programs *3dFWHMx* to estimate spatial smoothness and *3dClustSim* to estimate a minimum cluster-size threshold. For significant voxels, effect sizes were reported as z-scored BSR values. For dMRI parameters with significant effects, post-hoc summaries of group effects were obtained by averaging over significant voxels and refitting a GLM, with reporting of effects and bootstrapped 95%CIs.

### 2.5 Association of emotional health with dMRI data in COVID-19

To evaluate COVID-related associations between dMRI data and the three emotional health summary scores, a regression-weighted composite score was constructed that most strongly differentiated between COVID-19 and control groups. This approach was chosen to examine the relationship between dMRI parameters and emotional health for individuals with PACS, while avoiding excessive analysis of potentially redundant variables – analyses instead focused on the single predictor that encodes the most information about COVID-19 status. Composite scoring was achieved via linear discriminant analysis, regressing COVID-19 status (binary) onto the summary scores. All possible subsets of the three summary scores (SocSat, WelBei, NegAff) were examined as predictors of COVID-19 status, and the resulting models were compared using the Akaike information criterion with small sample correction (AICC) (35). The optimal discriminant model was identified that minimized the AICC score, and performance was measured relative to other models in terms of the difference scores (ΔAICC) and relative likelihood values (LAICC). See Appendix 2 for full model comparison details, along with the results of variable importance testing.

The set of participant scores produced by the linear discriminant model were retained as the linear composite score that optimally discriminated between COVID-19 and control groups. Effect sizes were reported for this NIH composite score, in terms of the mean group difference, bootstrapped 95%CI, BSR and *p*-value. To assess the relationship between this score and post-COVID symptoms, it was correlated against individual symptoms within the COVID-19 group, for “ongoing” status only and for “combined” status (ongoing plus resolved). The composite was also correlated against total “ongoing” and “combined” symptom counts. Spearman correlation coefficients were calculated, with bootstrapped 95%CIs and *p*-values, and significance was assessed at a false discovery rate (FDR) threshold of 0.05, in order to control for multiple comparisons.

To assess the relationship between the emotional sequelae and post-COVD brain physiology, voxel-wise bootstrapped partial correlations were then conducted, correlating dMRI measures against the NIH composite score. This was assessed within the COVID-19 group, with covariates adjusting for age and sex. Significant voxels were identified as in the previous section, based on 99.5%CIs adjusted for multiple comparisons at *p* < 0.05 using cluster-size thresholding. For significant regions, the effect sizes were again reported using z-scored BSR values. For dMRI parameters with significant effects, post-hoc summaries of effect sizes were obtained by averaging over significant voxels and recomputing the partial correlations of dMRI values with emotional composite scores, and bootstrapped 95%CIs.

## 3 Results

### 3.1 Analysis of clinical and demographic data

A total of 54 COVID-positive participants and 14 COVID-negative controls had been recruited and scanned. Table 1 shows that the groups had similar age ranges, proportions of male/female participants and years of education. None of the study participants had medical comorbidities, and none of the participants in the COVID-19 group had severe respiratory symptoms requiring medical intervention. In terms of symptoms, both groups showed moderately high, and largely comparable, reporting rates for individual symptoms, both ongoing and resolved, along with comparable total symptom counts. The only notable group difference in Table 1 was a higher prevalence of ongoing headache symptoms in the COVID-19 group.

**Table 1:** Summary of demographic and clinical data for study participants.

	Controls (*N* = 14)	COVID-19 (*N* = 54)	Group diff (SE)	Effect statistics	
				BSR	*p*-value
Years of Age, mean (SD)	40.8 (14.2)	40.9 (12.1)	0.1 (4.0)	0.03	0.986
Female, total (percent)	8/14 (57%)	36/53 (67%)	9.5 (14.5) %	0.65	0.495
Years of Education, mean (SD)	16.3 (2.4)	16.3 (2.2)	0.0 (0.7)	0.02	0.992
Days (symptom onset to scan)	194 [152, 235]	115 [75, 174]	−79 (29)	−2.67	0.011
Days (COVID test to scan)	151 [52, 235]	114 [71, 169]	−37 (42)	−0.89	0.361
Fever [ongoing] [resolved]	0/14 (0%) 8/14 (57%)	0/54 (0%) 37/54 (69%)	– 11 (15)	− 0.77	– 0.414
Cough [ongoing] [resolved]	2/14 (14%) 6/14 (43%)	8/54 (15%) 30/54 (56%)	1 (10) 13 (15)	0.05 0.84	0.858 0.374
Sore throat [ongoing] [resolved]	1/14 (7%) 7/14 (50%)	2/54 (4%) 38/54 (70%)	−3 (8) 20 (15)	−0.46 1.39	0.716 0.174
Shortness of breath [ongoing] [resolved]	4/14 (29%) 3/14 (21%)	13/54 (24%) 18/54 (33%)	−5 (13) 12 (13)	−0.34 0.93	0.741 0.368
Excess fatigue [ongoing] [resolved]	5/14 (36%) 5/14 (36%)	17/54 (31%) 32/54 (59%)	−4 (15) 24 (15)	−0.29 1.64	0.786 0.096
Gastrointestinal issues[ongoing] [resolved]	3/14 (21%) 3/14 (21%)	5/54 (9%) 25/54 (46%)	−12 (12) 25 (13)	−1.07 1.88	0.299 0.074
Issues with smell/taste[ongoing] [resolved]	1/14 (7%) 4/14 (29%)	12/54 (22%) 21/54 (39%)	15 (9) 10 (14)	1.68 0.75	0.122 0.453
Headache [ongoing] [resolved]	0/14 (0%) 1/14 (7%)	13/54 (24%) 10/54 (19%)	24 (8) 11 (9)	4.21 1.34	<0.001 0.184
Symptom count (ongoing)	2.1 (2.4)	2.1 (2.3)	0.0 (0.7)	0.01	0.989
Symptom count (combined)	4.9 (3.8)	6.7 (2.8)	1.8 (1.0)	1.79	0.077
Social satisfaction summary	41.3 (13.1)	45.9 (8.9)	4.6 (3.7)	1.25	0.202
Well-being summary	44.4 (7.9)	44.2 (6.9)	−0.2 (2.3)	−0.08	0.928
Negative affect summary	55.5 (12.4)	58.1 (8.6)	2.6 (3.6)	0.72	0.464

The mean and standard deviation (SD) are reported for normally-distributed data, while the median is reported with lower and upper distribution quartiles [Q1, Q3] for non-normal data, and the proportion and percentage are reported for categorical data. For between-group comparisons, the mean difference and bootstrapped standard error (SE) are reported, along with the z-scored bootstrap ratio (BSR) and a percentile-based *p*-value.

Examining NIH summary scores, moderately strong correlations were observed between SocSat and WelBei (mean and 95%CI: 0.52, [0.36, 0.66]), between SocSat and NegAff (−0.58, [−0.72, −0.41]), and between WelBei and NegAff (−0.75, [−0.84, −0.62]). In general, group means of the summary scores showed modest but significant differences from the normative mean (*T* = 50) in SocSat, for both COVID-19 (−4.1 [−6.3, −1.6], BSR = −3.33, *p* = 0.006) and controls (−8.7, [−15.5, −2.0], BSR = −2.59, *p* = 0.008); in WelBei, for both COVID-19 (−5.8, [−7.6, −3.9], BSR = −6.20, *p* < 0.001) and controls (−5.6 [−9.6, −1.3], BSR = −2.65, *p* = 0.009); and in NegAff for COVID-19 (+8.1 [+5.6, +10.5], BSR = 6.71 *p* < 0.001), although effects were non-significant for controls (+5.5 [−0.8, +12.3], BSR = 1.74, *p* = 0.074). The average summary scores, however, were not significantly different between COVID-19 and control groups, as noted in Table 1.

### 3.2 Effects of COVID-19 on dMRI data

**Figure 1:**
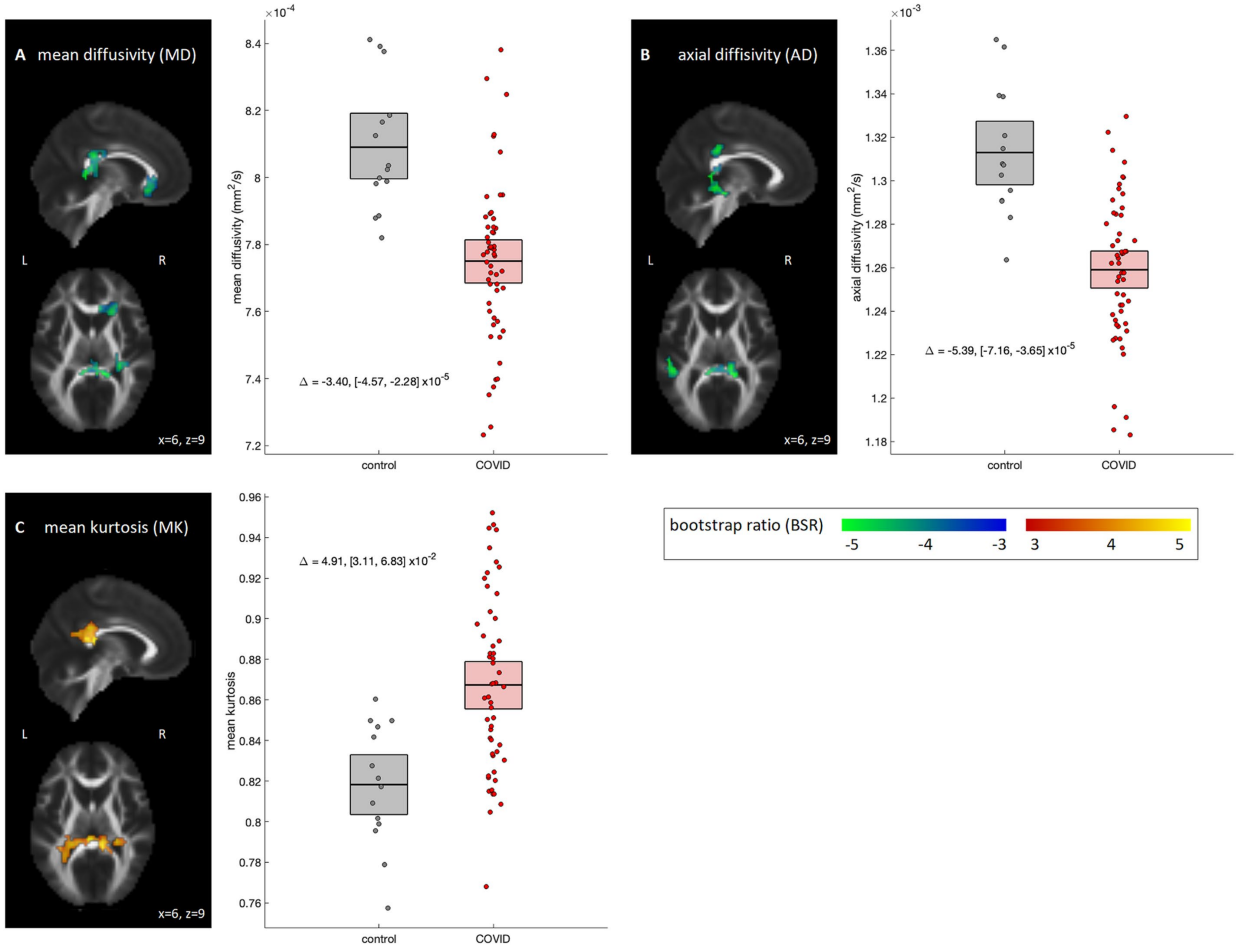
Diffusion MRI parameters showing significant differences between COVID-19 and control groups. Significant parameters include **(A)** mean diffusivity (MD), **(B)** axial diffusivity (AD) and **(C)** mean kurtosis (MK). For significant regions, standardized effect sizes are shown in z-score scale via bootstrap ratio (BSR) values, displayed as maximum intensity projections in the sagittal and axial planes, overlaid on mean FA slices from Montreal Neurological Institute (MNI) coordinates (*x* = 6, *z* = 9). Group differences are shown as scatter plots, depicting the mean value within significant voxels for each participant. Boxes denote the mean and bootstrapped 95% confidence interval (95%CI) of the mean for each group. Group differences are also displayed as text, with bootstrapped 95%CIs.

Voxel-wise analyses of the dMRI parameter maps are depicted in Figure 1. The COVID-19 group showed significant reductions in MD localized to the right anterior corona radiata (volume = 1701 mm^3^, center of mass = [21, 27, −9], peak BSR value = −4.24), splenium of the corpus callosum (1,350 mm^3^, [6, −39, 9], −5.62) and right posterior corona radiata (1,215 mm^3^, [30, −33, 21], −4.28) (Figure 1A); but no significant differences were observed for FA (95% interval of BSR values, computed over all voxels: [−1.59, 2.52]). Examining diffusion components, effects were significant for AD within the splenium of corpus callosum, including right superior (918 mm^3^, [15, −36, 30], −4.84) and medial aspects (918 mm^3^, [6, −39, 6], −7.09) and left sagittal stratum (1,161 mm^3^, [−48, −36, −12], −5.05) (Figure 1B), but non-significant for RD (95% interval of BSR values: [−2.74, 1.04]). Analyses of MK also found increased diffusion kurtosis in the medial aspect of splenium of corpus callosum (1863 mm^3^, [3, −36, 18], 4.54), left tapetum (1971 mm^3^, [−24, −42, 21], 4.03), and right posterior corona radiata (1,053 mm^3^, [30, −36, 24], 4.12) (Figure 1C). For the NODDI parameters, no significant group differences were identified for ODI, V_IC_ or V_ISO_ (95% intervals of BSR values: [−1.86, 2.37], [−0.86, 2.41] and [−2.41, 1.28], respectively). Post-hoc analyses within the identified clusters found that results were not significantly influenced by supplementary clinical/demographic factors including ongoing symptom burden, initial symptom burden or time from symptom onset to MRI scan (see Appendix 3 for details).

### 3.3 Association of emotional health with dMRI data in COVID-19

**Figure 2:**
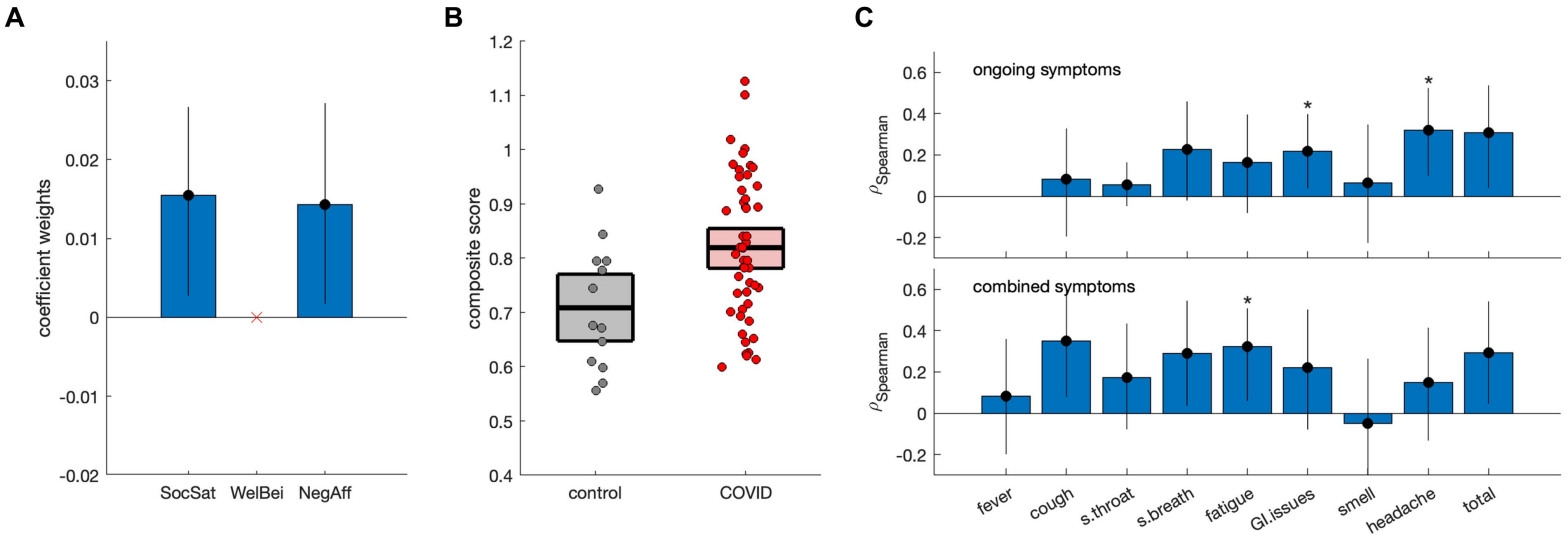
Discriminant analysis of NIH summary scores [social satisfaction (SocSat), well-being (WelBei) and negative affect (NegAff)] with respect to COVID status. **(A)** Weights of the coefficients of effect for a three-factor discriminant model, with error bars denoting bootstrapped 95% confidence intervals (95%CIs). **(B)** Individual participant scores for the new NIH composite score, plotted separately for control and COVID-19 groups. Horizontal lines denote group means and boxes indicate bootstrapped 95%CIs of the mean. **(C)** Spearman correlations of the NIH composite score with the presence of ongoing symptoms and combined symptoms (i.e., ongoing and resolved), respectively. Error bars denote bootstrapped 95%CIs of the mean, and ‘*’ indicates significant correlations at a False Discovery Rate (FDR) threshold of 0.05.

Results of discriminant analysis on the NIH summary scores are depicted in Figure 2. Although individual scores were non-significant in Table 1, model testing revealed that the SocSat+NegAff model yielded the best explanatory power, with “next-best” model SocSat+WelBei+NegAff having moderately less support (ΔAICC = 2.06, *L*_AICC,I_ = 35.6%) and all other models having substantially less support (ΔAICC ≥3.36, *L*_AICC,I_ ≤ 18.7%). Further testing affirmed that the SocSat+NegAff model best explained between-group differences (see Appendix 2 for details). As shown in Figure 2A, positive coefficient weights of comparable magnitude are obtained for SocSat (mean and 95%CI: 0.0158, [0.0027, 0.0267], BSR = 2.63, *p* = 0.012) and NegAff (0.0145, [0.0017, 0.0272], BSR = 2.18, *p* = 0.024). As shown in Figure 2B, this 2-variable model also produced elevated composite scores in the COVID-19 group (0.11, [0.04, 0.18], BSR = 3.11, *p* = 0.002), indicating higher relative values of both SocSat and NegAff for this group. In the COVID-19 group, positive correlations were also seen between the NIH emotional composite scores and the presence of viral symptoms (Figures 2C,D). At an FDR of 0.05, this included gastrointestinal issues (ongoing), headache (ongoing), and fatigue (combined). At an uncorrected *p* < 0.05, further effects were identified for cough (combined), shortness of breath (combined) and total symptoms (ongoing and combined).

**Figure 3:**
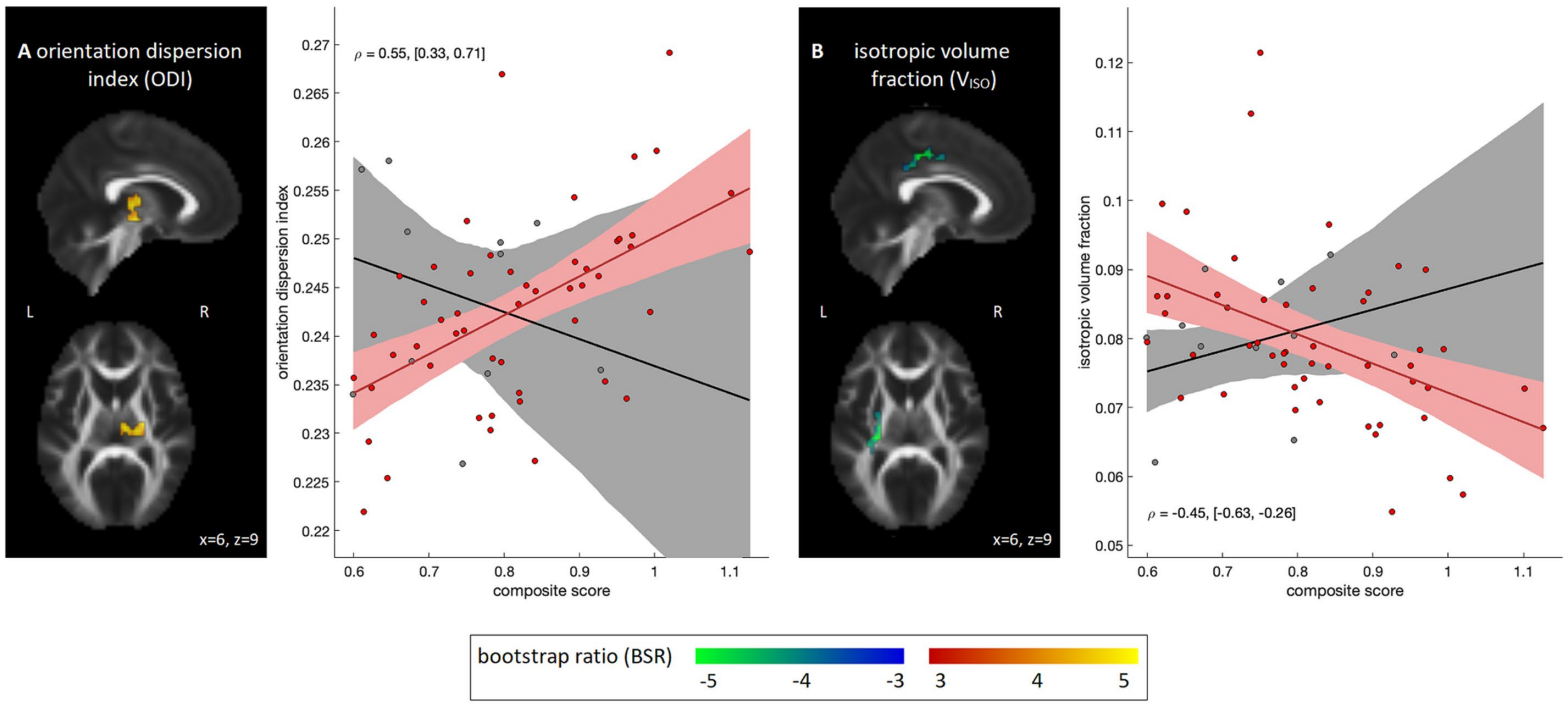
Diffusion MRI parameter maps showing significant associations with emotional composite score within the COVID-19 group; results for the control group are also shown for comparison purposes. Significant parameters include **(A)** orientation dispersion index (ODI) and **(B)** intracellular water volume fraction (V_IC_). For significant regions, standardized effect sizes are shown in z-score scale via bootstrap ratio (BSR) values, displayed as maximum intensity projections in the sagittal and axial planes, overlaid on mean FA slices from Montreal Neurological Institute (MNI) coordinates (*x* = 6, *z* = 9). Associations are shown as scatter plots, depicting the mean value within significant voxels for each participant. The line of best fit is plotted, with the bootstrapped 95% confidence interval (95%CI) depicted as a shaded area. Partial correlations are also displayed as text, with bootstrapped 95%CIs.

Voxel-wise analyses correlating diffusion parameter data with the NIH composite scores are depicted in Figure 3. Within the COVID-19 group, no significant associations were identified for DTI measures of FA, MD, AD and RD after applying cluster-size thresholding (95% intervals of BSR values: [−1.83, 2.25], [−2.45, 0.96], [−2.59, 1.16], and [−2.45, 1.17], respectively). Effects were similarly non-significant for MK. Among the NODDI measures, however, significant positive associations with ODI (Figure 3A) were seen within the right posterior limb of the internal capsule (1,296 mm^3^, [18, −21, 0], 4.50). In addition, significant negative associations with V_ISO_ were seen dorsally within the superior longitudinal fasciculus (volume = 1,052 mm^3^, center of mass = [−27, −24, 42], peak BSR value = 7.48), although effects for V_IC_ were non-significant (95% interval of BSR values: [−2.43, 1.66]). Post-hoc analyses within the identified clusters found that results were not significantly influenced by supplementary clinical/demographic factors including ongoing symptom burden, initial symptom burden or time from symptom onset to MRI scan (see Appendix 3 for details).

## 4 Discussion

This study found evidence of chronic differences in white matter microstructure among COVID-positive individuals with persistent symptoms, relative to COVID-negative individuals with persistent viral symptoms. For DTI metrics, lower MD and AD values were identified, indicating reduced water diffusion, mainly along the primary diffusion axis. Conversely, neither FA nor RD yielded significant effects, indicating unreliable effects of COVID-19 status on the radial component of diffusion. The findings of reduced AD and MD suggest increased axonal pathology in the COVID-19 group relative to controls (36), e.g., axonal swelling and cytoskeletal disruption. Conversely, the absence of RD and FA effects suggests a lack of support for altered myelination in the COVID-19 group (37).

These results are aligned with a study that compared individuals with PACS at 11 months post-infection (hospitalized and non-hospitalized) to uninfected controls (23). They also found reduced MD and AD, with affected regions including the corpus callosum and longitudinal fasciculus. Current results are also partially aligned with a study of hospitalized individuals with PACS at 3 months post-infection, which found reduced MD, AD and RD, and increased FA (16). They also partly align with a study of non-hospitalized individuals with PACS at 6 months post-infection, which reported reduced MD and increased FA in multiple white matter tracts (19). However, other studies have observed reduced FA and increased diffusivity, in both hospitalized (18) and non-hospitalized (17) cohorts, suggesting heterogeneous post-COVID microstructural changes. In addition, a large prospective study of mainly non-hospitalized individuals, imaged an average of 4–5 months post-infection, reported areas of increased diffusivity relative to pre-infection imaging (14). Effects were spatially heterogeneous in the brain though, with diffusivity reductions also seen in many areas, including the splenium of the corpus callosum, posterior corona radiata and sagittal stratum. Previous studies have consistently found significant dMRI effects in COVID-19 survivors relative to uninfected controls; the present study extends these findings by identifying significant effects in COVID-positive individuals with persistent symptoms relative to COVID-negative individuals with similar symptoms.

The DTI results in this study are complemented by increased MK, indicating a greater departure from unrestricted Gaussian diffusion of water in these regions for the COVID-19 group. By contrast, NODDI analyses showed no significant alterations in ODI, V_IC_ or V_ISO_. A previous study observed reduced V_IC_, albeit for a mixed cohort of hospitalized and non-hospitalized individuals, compared to uninfected controls (15). Furthermore, microstructural effects of the previous study were smaller in individuals with shorter hospital stays (15), supporting the lack of findings in this study. In the previously noted large prospective study (14), altered ODI and V_ISO_ were seen relative to pre-infection imaging, although the magnitude and direction of effects showed high spatial variability in the brain. Collectively, dMRI findings of the present study suggest that the microstructural environment of white matter is altered for the COVID-19 group, but without substantial differences in tissue water contributions. As noted for the DTI parameters, this may represent axonal pathology, or alternatively, an accumulation of cellular debris following neuroinflammation. Based on post-hoc testing, group differences do not appear to be influenced by initial or ongoing viral symptom burden, nor time from symptom onset to imaging, replicating prior literature in PACS cohorts (23).

The present study also identified an NIH toolbox composite score of emotional health that distinguished the COVID-19 group from controls. Analyses of SocSat, WelBei and NegAff in isolation found that mean values tended to be lower than the normative mean for both groups, with no significant differences between COVID-19 and control groups. Multivariate analysis, however, showed significant positive effects of SocSat and NegAff when both were in the model. This may be interpreted as the COVID-positive individuals experiencing greater negative affect (i.e., sadness, fear, anger) after adjusting for level of social satisfaction. The interpretation is supported by positive associations between the composite score and self-reported COVID symptoms, with significant effects relating to headache and fatigue. Despite the latter associations, group differences in symptoms were not seen, except for ongoing headache. This is consistent with many symptoms being non-specific signs of viral infection (e.g., fever, cough, sore throat). Furthermore, non-headache symptoms more prevalent in COVID-19 (e.g., fatigue, hyposmia, hypogeusia) (7) may have effects mitigated by the relatively mild sequelae in the present cohort. This should be explored in future research, with graded symptom scales providing more granular information that may better map onto NIH emotional scores.

Psychological effects are commonly reported in the acute phase of COVID-19 infection, including signs of depression, anxiety, and stress (38, 39). Among individuals with PACS, distress is similarly prevalent, often centered around a perceived lack of support and difficulty with daily activities (40) – such effects may be exacerbated by a persistent post-COVID inflammatory response (41). Interestingly, in the present cohort of non-hospitalized COVID-positive individuals, social satisfaction was not significantly lower than for controls. This highlights that COVID-negative individuals with viral symptoms may also have suffered from a perceived lack of support, given the widespread disruption of social systems during the pandemic (42–44), and that this should be acknowledged when characterizing mental health in COVID-19 research. The results provide evidence for a distinct pattern of emotional sequelae in COVID-positive individuals, compared to COVID-negative individuals with similar post-infection symptoms.

The study further identified significant correlations between dMRI parameters and the NIH emotional composite score in the COVID-19 group, supporting the presence of white matter abnormalities underlying the emotional sequelae. For DTI parameters and MK, no significant associations were identified. For the NODDI parameters, however, a significant positive association was seen with ODI, indicating greater dispersion of neurites within the identified regions. An increase in ODI has been linked to multiple chronic pathologies including demyelination (45), ischemic injury (46) and diffuse axonal injury (47), although the effects may also represent anatomical differences that predate COVID-19 infection. A negative association with V_ISO_ was also observed, indicating reduced extracellular free water within dorsal white matter. These results suggest accumulating intracellular water that is not neurite-specific, for individuals with prior COVID-19 infection and greater negative affect. Such results agree with animal models, in which neural injury is often accompanied by cellular swelling, glial hypertrophy and neuroplastic change, all of which may reduce extracellular free water (48). The present findings differ, however, from dMRI studies of chronic inflammation in other neurological conditions, which have reported increased free water (49, 50). A previous study of PACS also reported correlations with neuropsychiatric symptoms, albeit for standard DTI measures – they reported that FA of the sagittal stratum was correlated with fatigue, and MD of the amygdala was correlated with perceived stress (19). Despite the differing dMRI parameters and brain regions of interest, these results collectively support the interpretation that COVID-positive individuals endorsing higher emotional sequelae tend to have greater alterations in white matter microstructure.

Although significant effects of COVID-19 were identified for both clinical and neuroimaging indices, a discussion of study limitations is warranted. One such limitation is diagnostic uncertainty of the control group. While specificity of RT-PCR testing is high, studies have highlighted the often poor sensitivity of testing (51). Sensitivity is also time-dependent, with debate around the window of detectability (52). As such, there is a risk of false negatives in the control group, which may lead to under-estimating the effects of PACS on cerebral microstructure. This represents a challenge inherent to studies of COVID-19 with symptom-matched controls, and resolving the issue would require repeated RT-PCR testing conducted prospectively, or more restrictive selection criteria applied to COVID-negative controls. The sample sizes are also limited, particularly for the control group, although given the lack of data assessing differences in dMRI and emotional health data for COVID-19 with persistent symptoms, relative to non-COVID infection, this represents a unique dataset. The more common literature practise is to contrast individuals who have recovered from COVID-19 against uninfected controls. The present study focused on differences in symptomatic individuals with and without COVID-19 diagnosis. However, it is unclear to what extent dMRI values in the control group are altered by infection, and whether dMRI alterations in the COVID-19 group are specific to PACS. To this end, future research should include additional control groups, e.g., consisting of non-infected individuals and COVID-infected individuals without persistent symptoms. Another important factor to consider is whether the presence of specific symptom complaints moderates the relationship between dMRI and emotional symptoms in PACS. For example, issues related to olfaction and headache may represent distinct forms of neural injury and/or have differing effects on emotional well-being. This will be an important consideration to investigate in future research. The observed white matter effects on emotional health may also be exacerbated by other processes such as metabolic dysfunction (53), altered brain function (54) and abnormal perfusion (55); future work in PACS should develop more complete models that incorporate these different processes, informed by multi-modal MRI. Lastly regarding dMRI parameters, it is important to emphasize that “standard” parameter settings were used for the NODDI model, which may not optimally capture post-COVID microstructural changes (56). Further work is needed to investigate their validity in this group and potentially refine the NODDI model for improved sensitivity to PACS effects.

In conclusion, the principal study finding was significantly altered water diffusion in the white matter of self-isolating COVID-positive individuals with persistent symptoms; this included reduced diffusivity, increased diffusion kurtosis and neurite dispersion. Distinct emotional sequelae were also identified among COVID-positive individuals, of increased negative affect after adjusting for social satisfaction. Negative affect in the COVID-19 group was further associated with neurite dispersion and reduced free water in white matter. These findings provide new insights into the emotional and pathophysiological correlates of persistent post-COVID symptoms.

## Data availability statement

The datasets presented in this study can be found in online repositories. The names of the repository/repositories and accession number(s) can be found below: the datasets analyzed for this study can be found in the figshare data repository at: https://doi.org/10.6084/m9.figshare.26036245.

## Ethics statement

The studies involving humans were approved by the Sunnybrook Health Sciences Centre ethics board. The studies were conducted in accordance with the local legislation and institutional requirements. The participants provided their written informed consent to participate in this study.

## Author contributions

NC: Formal analysis, Methodology, Writing – original draft, Writing – review & editing. ER: Data curation, Methodology, Writing – review & editing. JC: Methodology, Writing – review & editing. AS: Methodology, Writing – review & editing. FG: Methodology, Writing – review & editing. MM: Methodology, Writing – review & editing. BL: Methodology, Writing – review & editing. IC: Methodology, Writing – review & editing. CH: Methodology, Writing – review & editing. SB: Methodology, Writing – review & editing. BM: Data curation, Methodology, Writing – review & editing. SG: Data curation, Methodology, Writing – review & editing. TS: Methodology, Writing – review & editing.

## Footnotes

^1^ https://fsl.fmrib.ox.ac.uk/fsl

^2^ dti-tk.sourceforge.net

^3^ nitrc.org/projects/noddi_toolbox
