# Supplementary material for: Effects of post-acute COVID-19 syndrome on cerebral white matter and emotional health among non-hospitalized individuals
Source: Front Neurol. 2024 Aug 6;15:1432450. doi: 10.3389/fneur.2024.1432450 (PMC11333225; doi:10.3389/fneur.2024.1432450)
Supplement: Supplementary file 1 [file Table_1.DOCX]

Supplementary Material

Playing the Blame Game: How Attribution of Responsibility Impacts Consumer Attitudes Towards Plastic Waste

Monica Mayer*, Patrice Kohl

*** Correspondence:** Corresponding Author: [monica.mayer@ucf.edu](mailto:monica.mayer@ucf.edu)

# Supplementary Data

**Consumer Responsibility Experimental Stimulus**

**Your plastic waste addiction is wrecking the environment**

Consumers buy and throw away millions of tons of plastic waste annually, with devastating impacts for the environment.

More than 330 million tons of plastic waste are thrown away every year. Most of that waste is burned, landfilled or dumped directly into the environment, where it accumulates in rivers and oceans.

[Image of woman drinking out of a plastic bottle]

**Figure 1.** We consume more than 13 billion plastic water bottles per year

Growing consumer demand for plastic, especially single-use plastics, is exacerbating an already out-of-control plastic waste crisis. If our demand continues unabated, plastic waste in oceans could quadruple by 2050.

[Image of plastic waste on a beach]

**Figure 2.** Plastic waste discarded by consumers has accumulated to alarming levels in our oceans and waterways

Consumer demand for plastic is devastating wildlife. Wild animals often mistake plastic waste as food or become entangled in it.

[Image of an aquatic turtle caught in plastic rings]

**Figure 3.** An aquatic turtle entangled in plastic rings, a consequence of consumer plastic demand and improper disposal

Unless consumers like you reduce their plastic consumption, our oceans will continue to be polluted and wildlife will continue to be harmed, victims of your reliance on plastic and disposable mentality.

**Manufacturer Responsibility Experimental Stimulus**

**Manufacturers are pushing plastics on the world, wrecking the environment**

Packaging and plastics manufactures create millions of tons of plastic waste annually, with devastating impacts for the environment.

More than 330 million tons of plastic waste are produced every year. Most of that waste is burned, landfilled or dumped directly into the environment, where it accumulates in rivers and oceans.

[Image of a belt in a manufacturing plant lined with plastic water bottles]

**Figure 1.** Manufacturers produce more than 13 billion plastic bottles per year

Growing industrial production of plastic, especially single-use plastics, is exacerbating an already out-of-control plastic waste crisis. If plastic production continues unabated, plastic waste in the oceans could quadruple by 2050.

[Image of plastic waste on a beach]

**Figure 2.** Plastic waste produced by manufacturers has accumulated to alarming levels in our oceans and waterways

Industry plastic production is devastating wildlife. Wild animals often mistake plastic waste as food or become entangled in it.

[Image of an aquatic turtle caught in plastic rings]

**Figure 3.** An aquatic turtle entangled in plastic rings, a consequence of manufacturer plastic production and improper disposal

Unless manufacturers reduce their plastic production, our oceans will continue to be polluted and wildlife will continue to be harmed, victims of industry reliance on plastic and disposable mentality.
