# Supplementary material for: Effects of post-acute COVID-19 syndrome on cerebral white matter and emotional health among non-hospitalized individuals
Source: Front Neurol. 2024 Aug 6;15:1432450. doi: 10.3389/fneur.2024.1432450 (PMC11333225; doi:10.3389/fneur.2024.1432450)
Supplement: Supplementary file 2 [file Table_2.DOCX]

**Appendix-2: modelling of NIH emotional health effects**

**METHODS**

With the aim of identifying an emotional health composite score that is most relevant to the COVID-19 group, the three NIH emotion toolbox summary scores of “social satisfaction” (SocSat), “well-being” (WelBei) and “negative affect” (NegAff) were evaluated as predictors in a linear discriminant model, i.e., multiple linear regression with COVID-19 status as the binary outcome variable. All possible subsets of three predictors were considered, giving 8 potential models (including an intercept-only model). The models were compared via Akaike information criterion with small-sample correction (AICC) ^14^. For linear regression with *K* parameters, sample size *N* and model likelihood *L*, this is defined as:

$$AICC=-2log(L)+2K+2K(K+1)/(N-K-1)$$

In model comparison, the optimal model minimizes AICC, which balances model fit to the data (i.e., high values of *L*) against model parsimony (i.e., low values of *K*) to ensure good generalizability. For the *i*^th^ model, performance is assessed in terms of difference from the optimal model, ${\Delta AICC}_{i}={AICC}_{i}-{AICC}_{min}$ and relative likelihood $L_{AICC,i}=e^{-{\Delta AICC}_{i}/2}$. A larger $\Delta AICC$ value indicates less support for the sub-optimal model; standard guidelines state that $\Delta AICC<2$ indicates comparable support between the models, $2<\Delta AICC<6$ indicates less support for the sub-optimal model, $6<\Delta AICC<10$ indicates extremely weak support for the sub-optimal model, and $\Delta AICC>10$ indicates that the sub-optimal model is highly implausible and can be discarded out of hand ^14^. A smaller $L_{AICC}$ value similarly indicates less support for the sub-optimal model, quantified in terms of the relative plausibility of using it as an alternative for the optimal one. These parameters were calculated for all of the tested models, with the median taken over 2000 bootstrap resamples to ensure a robust estimate of model quality. Similarly, the bootstrapping procedure allows for the construction of inter-quartile intervals, to better characterize the sampling variation of these parameters.

To further validate model selection, variable importance was assessed for the three NIH emotion toolbox summary scores, by testing the effects of deleting individual variables from the full model, in terms of the magnitude and reliability of change in regression coefficients for the remaining variables. This was evaluated separately for each variable within a paired-measures bootstrap resampling framework, with reporting of the mean changes in coefficient values, 95% confidence intervals (95%CIs), bootstrap ratios (BSRs) and p-values for the remaining variables. Further exploratory analyses tested the impact of deleting different combinations of variables from the discriminant model, obtaining the coefficient values and bootstrapped 95%CIs for all possible subsets of (SocSat, WelBei, NegAff).

**RESULTS**

Figure S1 plots median ΔAICC values for the different models under consideration. The 2-variable SocSat+NegAff model is most frequently selected as optimal (median ΔAICC and interquartile range: 0.0, [0.0, 1.0]; median *L*_AICC_ and interquartile range: 100.0%, [60.6%, 100.0%]), showing moderately strong evidence over the intercept-only “null” model (5.2, [2.2, 8.8]; 7.4% [1.2%, 33.0%]). The nearest competing model, SocSat+WelBei+NegAff shows a modest decrease in support (2.1, [1.3, 2.3]; 35.6% [32.0%, 51.0%]), while all other models show a more substantial decrease in support (all ΔAICC > 3.4, all *L*_AICC_ < 18.7%). It is also noted that the optimal model (SocSat+NegAff) and its nearest competing model (SocSat+WelBei+NegAff) show most reliable performance, with relatively narrow interquartile range bars.


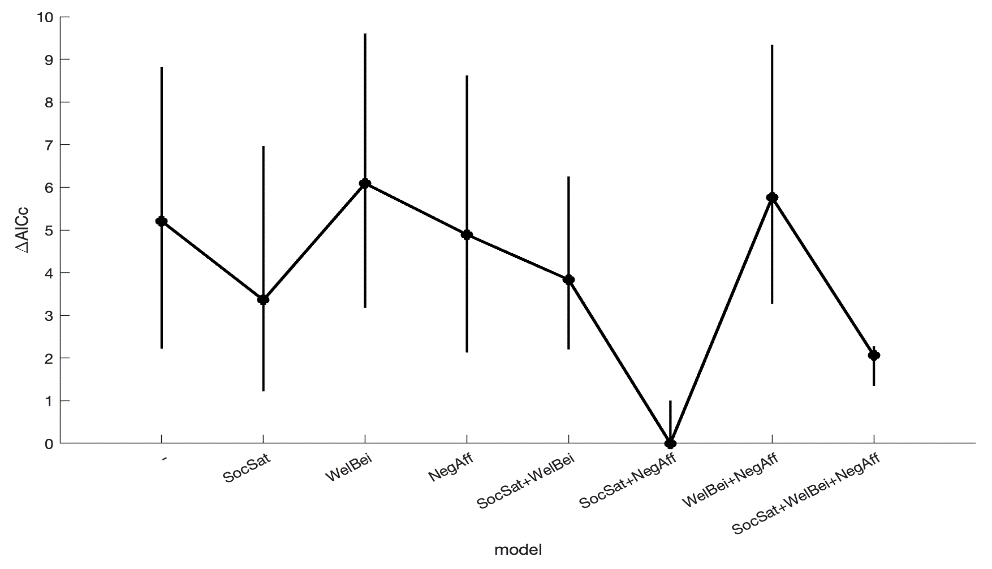


**Figure S1**: comparison of different discriminant models with NIH emotional composite scores, evaluated using the Akaike information criterion with small-sample correction (AICC). Each point represents the median difference in AICC for a given model, relative to the best-performing one (ΔAIC), taken over 2000 bootstrap resamples, along inter-quartile error bars. Models correspond to subsets of variables “social satisfaction” (SocSat), “well-being” (WelBei) and “negative affect” (NegAff), with “-“ denoting intercept-only model. The (SocSat+NegAff) model consistently minimizes the AICC and is seen in the main study results (Figure 1).

Bootstrap model testing further affirmed the joint importance of the SocSat and NegAff variables, as the removal of SocSat significantly decreased NegAff effects (Δ*b*=-0.007, 95%CI=[-0.016 -0.001], BSR=-1.79, p=0.020), but did not significantly impact WelBei (Δ*b*=0.004, 95%CI=[-0.002, 0.014], BSR=1.12, p=0.176). The removal of NegAff similarly decreased SocSat effects (Δ*b*=-0.004, 95%CI=[-0.010, -0.001], BSR=-1.65, p=0.046), and also altered WelBei (Δ*b*=-0.014, 95%CI=[-0.031 -0.001], BSR=-1.91, p=0.038), changing the coefficient from weakly positive to weakly negative. In contrast, the removal of WelBei had a minimal impact on the other variables (both |BSR|>-0.61, p>0.526).

The contributions of the NIH summary scores to the discriminant model are further explored in Figure S2, where coefficients and 95%CIs are shown for all possible subsets of (SocSat, WelBei, NegAff). Consistent with the univariate tests of Table 1, analysis using the individual summary scores in panels 1-3 show relatively weak effects and 95%CIs overlapping zero. Sub-models containing SocSat+WelBei and WelBei+NegAff show only slightly increased coefficients and 95%CIs overlapping zero, whereas SocSat+NegAff show significant effects, with similar results seen in the full model. This overall indicates the importance of including both SocSat and NegAff to obtain reliable associations with COVID-19 status, whereas the contributions of WelBei are more limited.


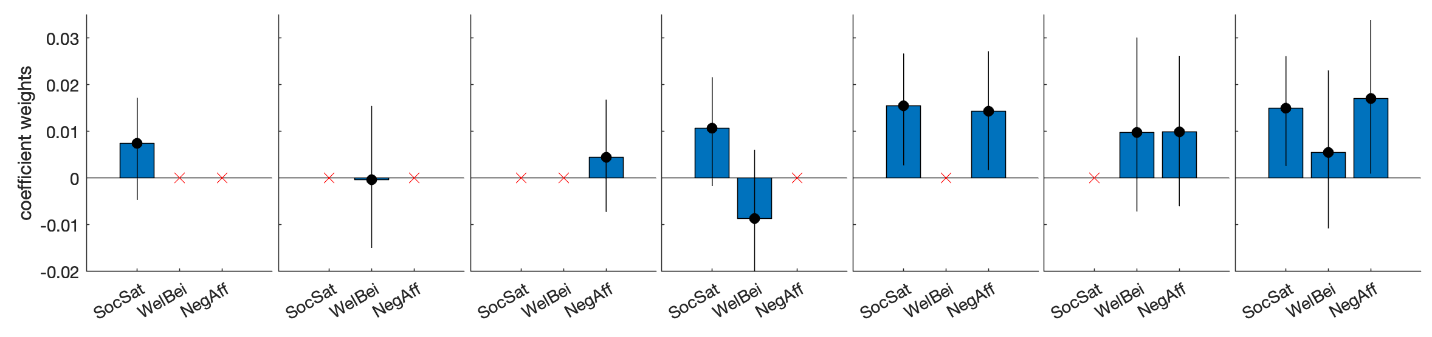


**Figure S2**: comparison of coefficients for different discriminant models with NIH emotional composite scores. Each panel shows coefficient weights with errorbars denoting bootstrapped 95% confidence intervals (95%CIs) of variables for a given model, in which excluded variables are denoted by a red “X”. Models correspond to different subsets of variables “social satisfaction” (SocSat), “well-being” (WelBei) and “negative affect” (NegAff). The fifth panel (SocSat+NegAff) is the model consistently minimizing the AICC criterion and is seen in the main results (Figure 1).
