# Supplementary material for: Effects of post-acute COVID-19 syndrome on cerebral white matter and emotional health among non-hospitalized individuals
Source: Front Neurol. 2024 Aug 6;15:1432450. doi: 10.3389/fneur.2024.1432450 (PMC11333225; doi:10.3389/fneur.2024.1432450)
Supplement: Supplementary file 3 [file Table_3.DOCX]

**Appendix-3: impact of supplemental clinical variables**

**METHODS**

A series of supplemental analysis models examine the impact of incorporating supplemental clinical variables of interest (VoI) into the main dMRI analyses, in terms of both their explanatory power, and their impact on the main effect of interest. The examined clinical VoI included: the number of “ongoing” self-reported symptoms (i.e., assessing the effects of viral symptom at time of imaging); the “combined” number of self-reported symptoms, both ongoing and resolved (i.e., assessing the effects of initial viral symptom burden); and days from symptom onset to MRI scan (i.e., measuring potential time-evolving recovery processes).

**Effects of COVID-19 on dMRI data**

For dMRI parameters with significant group effects, VoIs were tested by refitting GLMs on the mean dMRI values averaged over all significant voxels, for participants in both COVID-19 and control groups. First, the original model (COVID-19 status + age +sex) was refitted without/with each VoI included as a covariate, and the resulting change in the coefficient of effect for COVID-19 status was estimated. Second, the expanded model (COVID-19 status + age +sex + VoI) was fitted and the coefficient of effect for the VoI estimated. In both cases, bootstrap resampling was conducted, with the resulting BSRs and p-values reported. For these analyses, days from symptom onset to scan was log-transformed as the distribution in the combined COVID-19 and control groups deviated from normality (skew=1.4, kurtosis=5.6).

**Associations of emotional health with dMRI data in COVID-19**

For dMRI parameters with significant group effects, VoIs were tested by refitting partial correlation models on the mean dMRI values averaged over all significant voxels, for participants in the COVID-19 group. First, the original model (emotional composite score, adjusting for age +sex) was refitted without/with also controlling for the VoI, and the resulting change in the coefficient of partial correlation for the emotional composite score was estimated. Second, a partial correlation model of the VoI, adjusting for emotional composite score + age +sex was fitted and the coefficient of partial correlation for the VoI estimated. In both cases, bootstrap resampling was conducted and the resulting BSRs and p-values reported. For these analyses, days from symptom onset to scan was left untransformed, as the distribution within the COVID-19 group did not deviate substantially from normality (skew=0.7, kurtosis=2.8).

**RESULTS**

**Effects of COVID-19 on dMRI data**

Post-hoc regression analyses of the combined control and COVID-19 groups within the identified clusters found no significant associations of the dMRI parameter values with supplementary variables, including ongoing symptom burden, initial symptom burden or time from symptom onset to MRI scan (all |BSR|<1.15, p>0.239); nor did the inclusion of these variables within the models significantly alter the estimated effects of COVID-19 status on dMRI parameters (all |BSR|<1.05, p>0.240).

**Associations of emotional health with dMRI data in COVID-19**

Post-hoc partial correlation analyses of COVID-19 group within the identified clusters found that for ODI, there were significant positive associations with days from symptom onset to MRI scan (ρ=0.38, [0.10, 0.61], BSR=2.86, p=0.006), although the inclusion of this variable did not significantly alter the estimated effect of the emotional composite score (BSR=0.68, p=0.482), suggesting an approximately independent additive effect. For all other analyses of dMRI parameters and supplementary variables, correlations were non-significant (all |BSR|<1.35, p>0.175), nor did the inclusion of these variables within the models significantly alter the estimated associations with the emotional composite score (all |BSR|<0.84, p>0.302).
